# Efficacy and safety of neurokinin-1 receptor antagonist on cough suppression: a systematic review and meta-analysis

**DOI:** 10.3389/fphar.2026.1915023

**Published:** 2026-10-01

**Authors:** Ye Yang, Ji Wang, Zhiqiang Hu, Gang Wang, Junjie Li

**Affiliations:** 1 Department of Medical Oncology, Sichuan Clinical Research Center for Cancer, Sichuan Cancer Hospital & Institute, Sichuan Cancer Center, Affiliated Cancer Hospital of the University of Electronic Science and Technology of China, Chengdu, China; 2 Department of Respiratory and Critical Care Medicine, Clinical Research Center for Respiratory Disease, West China Hospital, Sichuan University, State Key Laboratory of Respiratory Health and Multimorbidity, Sichuan University, Laboratory of Pulmonary Immunology and Inflammation, Frontiers Science Center for Disease-related Molecular Network, Sichuan University, Chengdu, China; 3 Department of Pharmacy, Sichuan Clinical Research Center for Cancer, Sichuan Cancer Hospital and Institute, Sichuan Cancer Center, Affiliated Cancer Hospital of the University of Electronic Science and Technology of China, Chengdu, China; 4 Department of Breast Surgery, Sichuan Clinical Research Center for Cancer, Sichuan Cancer Hospital and Institute, Sichuan Cancer Center, Affiliated Cancer Hospital of the University of Electronic Science and Technology of China, Chengdu, China

**Keywords:** antitussive agents, cough, meta-analysis, neurokinin-1 receptor antagonist, urge to cough

## Abstract

**Introduction:**

The efficacy and safety of neurokinin-1 (NK-1) receptor antagonist in adults with cough remain unclear. Our objective was to systematically assess the efficacy and safety of NK-1 receptor antagonist for cough treatment using the latest available clinical evidence.

**Methods:**

A comprehensive literature retrieval was conducted across PubMed, Embase (via Ovid), the Cochrane Central Register of Controlled Trials (CENTRAL), and the ClinicalTrials.gov registry, encompassing data from database inception through 10 March 2026. This search aimed to identify randomized controlled trials (RCTs) assessing the therapeutic utility of NK-1 receptor antagonists in cough management. The abstracts and full-text articles were screened based on predefined inclusion and exclusion criteria. Meta-analysis was performed using R software (V 4.4.2). Main outcomes included the cough visual analog scale (VAS), awake objective cough frequency, urge to cough scale, 24-h cough frequency, and cough-specific patient-reported scale. Adverse events were also pooled.

**Results:**

Five RCTs comprising a total 719 participants were included, evaluating three NK-1 receptor antagonists: aprepitant, orvepitant, and serlopitant. Compared to placebo, NK-1 receptor antagonists demonstrated significant antitussive efficacy by improving cough VAS scores (mean difference [MD] = −6.90, 95 CI (−10.90, −2.90), p < 0.0001) and cough-specific patient-reported scale (standardized mean difference [SMD] = 0.50, 95 CI (0.22, 0.78), p < 0.0001). Notably, the treatment group experienced a significant reduction in the urge to cough scale (SMD: −0.31, 95% CI (−0.40, - group (RR: 1.33, 95% CI (1.15, 1.53), p < 0.0001).No significant difference was detected at the overall effect estimates of objective cough frequency.

**Conclusion:**

NK-1 receptor antagonists provide modest improvements in patient-reported subjective cough outcomes, but show no statistically significant reduction in objective cough frequency. Given the small evidence base, clinical heterogeneity among pooled conditions, and higher incidence of mild adverse events, conclusions regarding their clinical benefit must be drawn with caution. Large-scale, high-quality clinical studies are required to confirm their efficacy and safety.

## 1 Introduction

Cough, as an important defensive reflex, has a protective effect on the body (Morice et al., 2020). Nevertheless, persistent and abnormally severe coughing remains a widespread and debilitating condition, with an estimated prevalence of 5%–10% among the adult demographic (Song et al., 2015). In severe instances, this condition profoundly diminishes a patient’s overall wellbeing by triggering physical complications such as urinary incontinence, fainting episodes, and vocal impairment; these physical burdens frequently precipitate adverse psychosocial outcomes, including clinical depression, interpersonal strain, and societal withdrawal (Brignall et al., 2007; Chamberlain et al., 2015; Dicpinigaitis, 2021; Bali et al., 2024).

Although the current 2019 European Respiratory Society (ERS) guidelines provide a systematic framework of sequential diagnostic therapeutic trials for the management of chronic cough (Morice et al., 2020), significant academic controversies and severe challenges persist in clinical practice. On one hand, empirical therapies targeting peripheral pathways, such as anti-inflammatory agents (e.g., inhaled corticosteroids, ICS) and anti-reflux medications (e.g., proton pump inhibitors, PPIs), exhibit high therapeutic heterogeneity in unselected (unphenotyped) patient populations. Furthermore, prolonged or high-dose administration of these agents carries non-negligible risks of systemic adverse effects, including localized fungal infections associated with ICS (Calverley et al., 2003), as well as osteoporosis and gut dysbiosis associated with long-term PPI use (Giuliano et al., 2012; Moayyedi and Leontiadis, 2012). On the other hand, neuromodulators (e.g., low-dose morphine, gabapentin, and pregabalin) that target central and peripheral cough hypersensitivity states can elevate the cough threshold and desensitize the cough reflex to some extent (Morice et al., 2006; Ryan et al., 2012; Vertigan et al., 2016); however, they present a profound clinical dilemma. This predicament is driven by their high drop-out rates, the substantial proportion of patients forced to discontinue therapy due to intolerable central nervous system toxicities (Zaccara et al., 2017).

Currently, available treatment options for refractory chronic cough are limited. Traditional antitussive drugs, such as opioids, carry risks of addiction and respiratory depression, while non-opioid drugs like dextromethorphan are insufficiently effective and may cause intolerable side effects with long-term use. Consequently, the discovery of innovative, highly efficacious, and well-tolerated antitussive interventions represents a critical unmet clinical imperative (Smith, 2023). Fortunately, profound recent advances in elucidating the central and peripheral sensitization pathways of the cough reflex have established a robust mechanistic framework for the design of novel targeted pharmacotherapies (Mazzone and McGarvey, 2020).

Substance P serves as a pivotal neuromodulator in the pathogenesis of the cough reflex, exerting its effects via a dual mechanism. Peripherally, the interaction between Substance P and neurokinin-1 (NK-1) receptors on the airway epithelium precipitates the secretion of pro-inflammatory cytokines (Zhang et al., 2026). Centrally, the ligation of this peptide to NK-1 receptors situated on brainstem neurons facilitates the neural transmission required to elicit a cough (Badri and Smith, 2019; Smith et al., 2021). Consequently, pharmacological blockade of these pathways using NK-1 receptor antagonists has emerged as a promising antitussive strategy (Noronha et al., 2020; Smith et al., 2021). Clinical evidence supports this paradigm: multiple randomized controlled investigations, including double-blind and crossover designs, have demonstrated that aprepitant substantially attenuates cough burden in patients with advanced lung cancer (Noronha et al., 2020; Smith et al., 2021). Furthermore, parallel studies evaluating orvepitant in cohorts with refractory chronic cough reported durable enhancements in patient quality of life, alongside marked reductions in both subjective severity via the Visual Analogue Scale (VAS) and objective cough frequency (Smith et al., 2019).

Although preliminary clinical trials of NK-1 receptor antagonists show great promise in overcoming current therapeutic challenges in chronic cough (Smith et al., 2019; Noronha et al., 2020; Smith et al., 2021), existing findings remain fragmented. Most randomized controlled trials (RCTs) to date have been limited by small sample sizes, heterogeneous patient populations (e.g., refractory chronic cough versus lung cancer-associated cough), and inconsistent efficacy endpoints, making it difficult to draw definitive, uniform conclusions regarding their overall efficacy and safety. Because there was a lack of comprehensive, high-quality, evidence-based quantitative syntheses for this specific class of drugs, we conducted this systematic review and meta-analysis of all available RCTs to systematically evaluate the clinical efficacy, and safety of NK-1 receptor antagonists in the treatment of cough.

## 2 Methods

The methodology of this systematic evaluation strictly adhered to the Preferred Reporting Items for Systematic Reviews and Meta-Analyses (PRISMA) guidelines, Its prespecified review protocol was prospectively registered on the PROSPERO database(registration number: CRD42022311307) prior to data extraction and analysis.

### 2.1 Searches strategy

We searched the data in PubMed, Embase (via Ovid), Cochrane Central Register of Controlled Trials (CENTRAL), and ClinicalTrials.gov from their inception date to 10 March 2026, the date on which the final search was performed. Search terms included ‘Cough’ AND ‘Neurokinin-1 Receptor Antagonists’. Our search strategy is provided in Supplementary Material. To supplement the primary electronic retrieval, we manually cross-referenced the bibliographies of pertinent existing systematic reviews. Following the elimination of redundant citations, a two-tiered screening protocol was executed. Initially, two independent investigators evaluated the titles and abstracts of all retrieved records against predefined eligibility parameters. Subsequently, the full-text articles of tentatively qualified studies underwent rigorous independent scrutiny by the same pair of researchers. Any interpretative discrepancies emerging during these selection phases were resolved through arbitration by a third author.

### 2.2 Inclusion and exclusion criteria

Eligibility for this systematic review was established applying the standard PICOS methodology (Liberati et al., 2009). Included studies were required to be randomized controlled trials (RCTs), encompassing published or unpublished parallel, crossover, or cluster designs—that evaluated placebo-controlled interventions of NK-1 receptor antagonists in patients suffering from cough. Exclusion criteria comprised non-randomized trial designs, acute (single-dose) study models, insufficient methodological descriptions regarding the targeted drug or treatment duration, and the absence of requisite statistical variance data (means and standard deviations) that could not be resolved through author correspondence. Utilizing these predefined boundaries, two researchers independently executed a multi-stage eligibility screening, comprehensively evaluating the titles, abstracts, and full texts of the identified citations.

### 2.3 Data extraction

Data extraction was independently executed by two investigators, capturing bibliometric details (including author names, article titles, publication years, and journal identities), cohort sizes, baseline demographic profiles (such as age and gender distribution), assigned pharmacological interventions (summarized in Table 1), and prespecified clinical outcomes. To address inconsistencies in reported variance, standard deviations (SDs) were mathematically derived from standard errors (SEs) utilizing the established equation: standard deviation (SD) = standard error (SE) × √n. In scenarios where neither metric was explicitly documented, SD approximations were imputed from available confidence intervals, t-statistics, interquartile ranges, data spans, or exact p-values, strictly adhering to the imputation algorithms outlined in Section 7.7.3 of the Cochrane Handbook for Systematic Reviews of Interventions. When these statistical derivation strategies proved insufficient, direct email correspondence was initiated with the corresponding authors to solicit the requisite raw datasets.

**TABLE 1:** Characteristics of the included studies.

Study	Study design	Mean age	Sample size (Female/male)	Disease	Intervention	Outcomes	Quality of literature
NCT03282591 (2019)	RCT	EG: 62.7 ± 11.21 Placebo: 62.6 ± 10.09	EG: 88 (20/68) Placebo: 88 (21/67)	Chronic refractory cough	5 mg serlopitant tablets, 12 weeks	Effectiveness: ①, ②, ③ Safely: ⑦, ⑧	low risk
NCT02993822 (2022)	RCT	EG1: 62.29 ± 9.85 EG2: 59.86 ± 13.173 EG3: 59.57 ± 10.02 Placebo: 62.24 ± 11.815	EG1: 79 (65/14) EG2: 78 (69/9) EG3: 79 (60/19) Placebo: 79 (59/20)	Refractory chronic cough	EG1: 10 mg orvepitant, 12 weeks EG2: 20 mg orvepitant, 12 weeks EG3: 30 mg orvepitant, 12 weeks	Effectiveness: ①, ②, ④-2, ⑥ Safely: ⑦, ⑧	low risk
Noronha et al. (2020)	RCT	EG: 53 ± 12.5 Placebo: 52 ± 14	EG: 64 (20/44) Placebo: 64 (25/39)	Advanced lung cancer	Aprepitant 125 mg D1, 80 mg D2 to D7	Effectiveness: ①, ④-1 Safely: ⑦, ⑧	High risk
Smith et al. (2021)	Randomized crossover trial	66 ± 6.74	20 (12/8)	Lung cancer	Aprepitant 125 mg day 1, 80 mg day 2–3	Effectiveness: ①, ②, ③, ④-1,⑤ Safely: ⑧	low risk
NCT05185089 (2025)	Randomized crossover trial	71.9 ± 7.17	80 (21/59)	Idiopathic pulmonary fibrosis	EG1: 10 mg orvepitant, 4weeks EG2: 30 mg orvepitant, 4weeks	Effectiveness: ①, ②, ③, ④-2, ⑤, ⑥ Safely: ⑦, ⑧	Low risk

① cough visual analog scale (VAS), ② awake objective cough frequency, ③ 24 h cough frequency, ④ cough-specific patient-reported scale (④-1 manchester cough in lung cancer scale, ④-2 Leicester Cough Questionnaire)⑤ Sleep cough frequency, ⑥ Urge to cough scale, ⑦ severe adverse events, ⑧ other (not including serious) adverse events. EG, Experimental Group.

### 2.4 Outcomes

This review focused on effectiveness and adverse event. The outcomes of effectiveness included visual analog scale (VAS), awake objective cough frequency, 24 h cough frequency, and cough-specific patient-reported scale (Manchester cough in lung cancer scale, Leicester Cough Questionnaire); For adverse events we evaluated separately severe adverse events and other (not including serious) adverse events.

### 2.5 Risk of bias assessment

Methodological quality and potential biases of the included trials were independently appraised by a pair of investigators utilizing the revised Cochrane risk-of-bias tool (RoB 2). The assessment framework systematically scrutinized five core domains: the integrity of the randomization process, any departures from the intended pharmacological interventions, the handling of missing outcome data, the validity of outcome measurements, and the potential for selective outcome reporting. Based on predefined algorithmic criteria, each study was ultimately categorized into one of three risk tiers: “low risk,” “some concerns,” or “high risk.” Any interpretative discrepancies arising between the primary assessors during this evaluation were reconciled either through mutual consensus or via formal adjudication by a third independent researcher.

### 2.6 Quality of the evidence

Two independent reviewers evaluated the strength of evidence for each outcome using the Grading of Recommendations Assessment, Development, and Evaluation (GRADE) framework (Guyatt et al., 2008). Initially, evidence was rated as high quality and subsequently downgraded based on the following factors: (1) publication bias, indicated by at least 10 studies and an Egger test p-value <0.05 (Ioannidis and Trikalinos, 2007); (2) imprecision, when fewer than 400 subjects were included in the meta-analysis(Mueller et al., 2007); (3) a high risk of bias in over 25% of the studies or some concerns in more than 50% of the studies (Mascarenhas et al., 2021); and (4) substantial heterogeneity, defined as I^2^ > 50% (Higgins and Green, 2008). The final ratings were summarized into one of four GRADE confidence levels: very low, low, moderate, or high (Brozek et al., 2009).

### 2.7 Statistical analysis

We will use the ‘metafor’ package in the R software (V 4.4.2) to perform a meta-analysis. For continuous outcomes, the effect sizes measure chooses the mean difference (MD) or standardized mean difference (SMD) of the change score (end point minus baseline score) according to whether the unit or scale is the same. The change in baseline and ending values for both groups was computed as: $Mean_{change}=Mean_{ending}-Mean_{baseline}$ ; $SD_{change}=\sqrt{SD_{baseline}^{2}+SD_{ending}^{2}-2*R*SD_{baseline}*SD_{ending}}$ , where R is a constant (R = 0.5) (McKenzie and Brennan, 2019). For binary outcomes, the effect sizes measure chooses the relative risk (RR). Quantitative synthesis of the extracted effect sizes was conducted utilizing appropriate statistical frameworks. Specifically, a random-effects model was adopted when substantial inter-study heterogeneity was detected (I^2^ > 50% and p ≤ 0.10); conversely, a fixed-effects approach was applied under homogeneous conditions. Pooled estimates (including mean difference [MD], standardized mean difference [SMD], or risk ratio [RR] and their 95% confidence intervals [CI]) were visualized using forest plots. To formally quantify between-trial variance, the I^2^ statistic (ranging from 0% to 100%) was employed. In accordance with established guidelines, I^2^ thresholds of <25%, 25%–50%, and >75% were indicative of low, moderate, and severe heterogeneity, respectively, with any value surpassing 50% strictly classified as substantial. Furthermore, pooled statistical analyses for specific adverse events were executed only if they were documented in at least two independent trials. Finally, given the restricted pool of included literature (five trials, well below the conventional 10-study prerequisite), we refrained from evaluating publication bias. Sensitivity analyses were performed by excluding the study with a high risk of bias to evaluate the robustness of the primary outcomes.

## 3 Results

The sequential literature retrieval and screening workflow is elucidated in Figure 1. Our initial search of four electronic databases retrieved 765 published studies. After removing duplicates and screening titles and abstracts, 40 citations were secured for comprehensive full-text evaluation. Through rigorous in-depth scrutiny by three independent pairs of investigators to verify targeted clinical endpoints, the literature pool was ultimately distilled to five eligible trials. These studies contributed an aggregate cohort of 719 subjects, comprising 410 females (57.02%) and 309 males (42.98%). Individual trial cohorts varied in size from 20 to 315 enrollees, presenting an overall mean age of 61.22 years (summarized in Table 1). Regarding methodological rigor, the integrated literature demonstrated a broadly robust profile with a low risk of bias, barring the singular trial by Noronha et al. (2020), which was designated as high risk (comprehensive quality metrics are detailed in Table 2).

**FIGURE 1:**
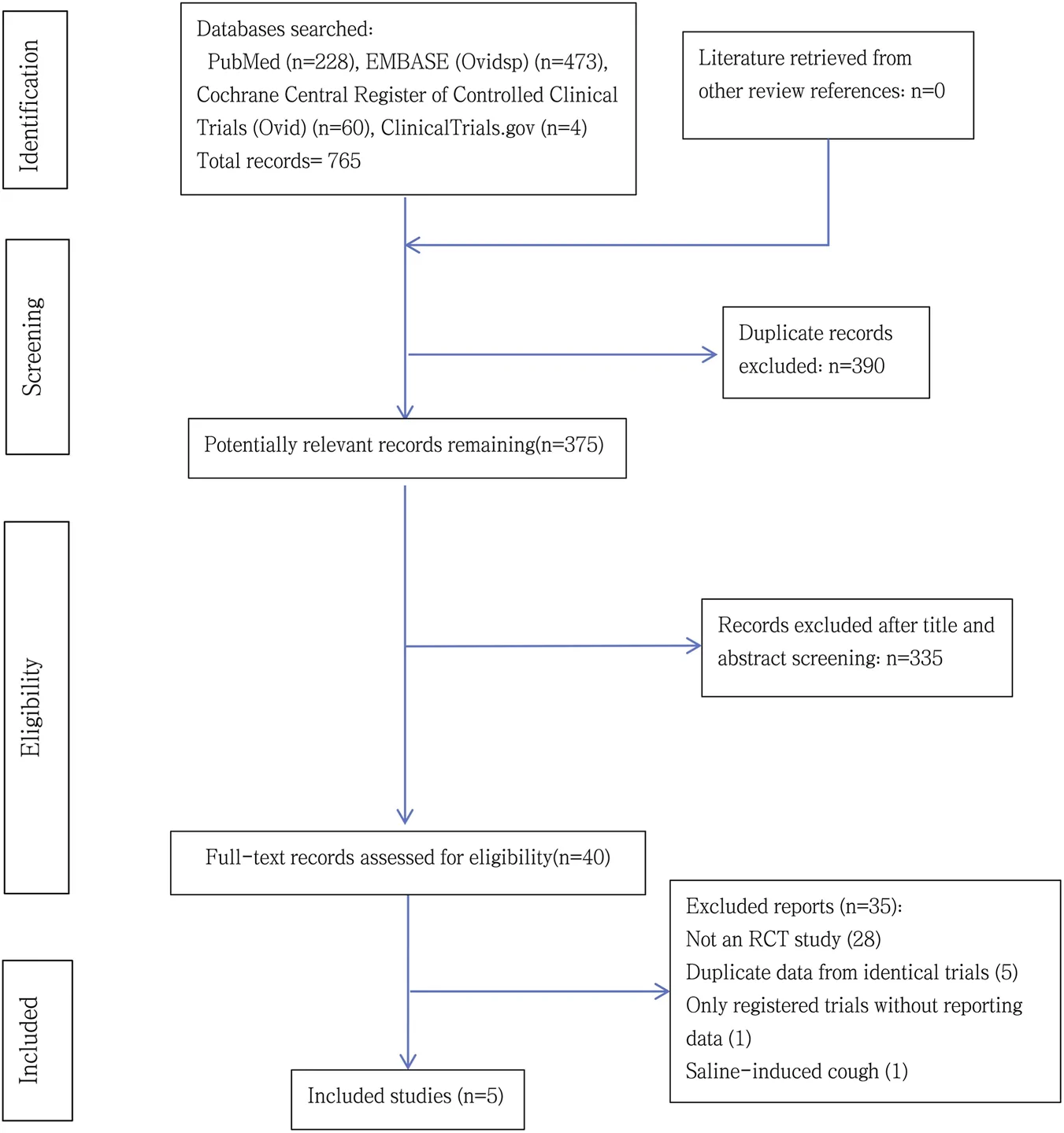
PRISMA Flow diagram of the search process for studies.

**TABLE 2:** The risk of bias assessment for the individual included studies.

Study	Randomization process	Deviations from intended interventions	Missing outcome data	Measurement of the outcome	Selection of the reported result	Overall bias
NCT03282591 (2019)	Low	Low	Low	Low	Low	Low
NCT02993822 (2022)	Low	Low	Low	Low	Low	Low
Noronha et al. (2020)	Low	High	Low	Low	Low	High
Smith et al. (2021)	Low	Low	Low	Low	Low	Low
NCT05185089 (2025)	Low	Low	Low	Low	Low	Low

### 3.1 Effectiveness

#### 3.1.1 Cough visual analog scale (VAS)

Five studies with a total of 38 MDs assessed the VAS scale. Compared with those having placebo, adults with cough receiving NK-1 receptor antagonists had a significantly reduced VAS which reflects the severity of the cough (MD: −6.90, 95 CI (−10.90, −2.90), *p* < 0.0001) The heterogeneity was substantial (I^2^ = 56.18% and *p* = 0.0033) (Figure 2).

**FIGURE 2:**
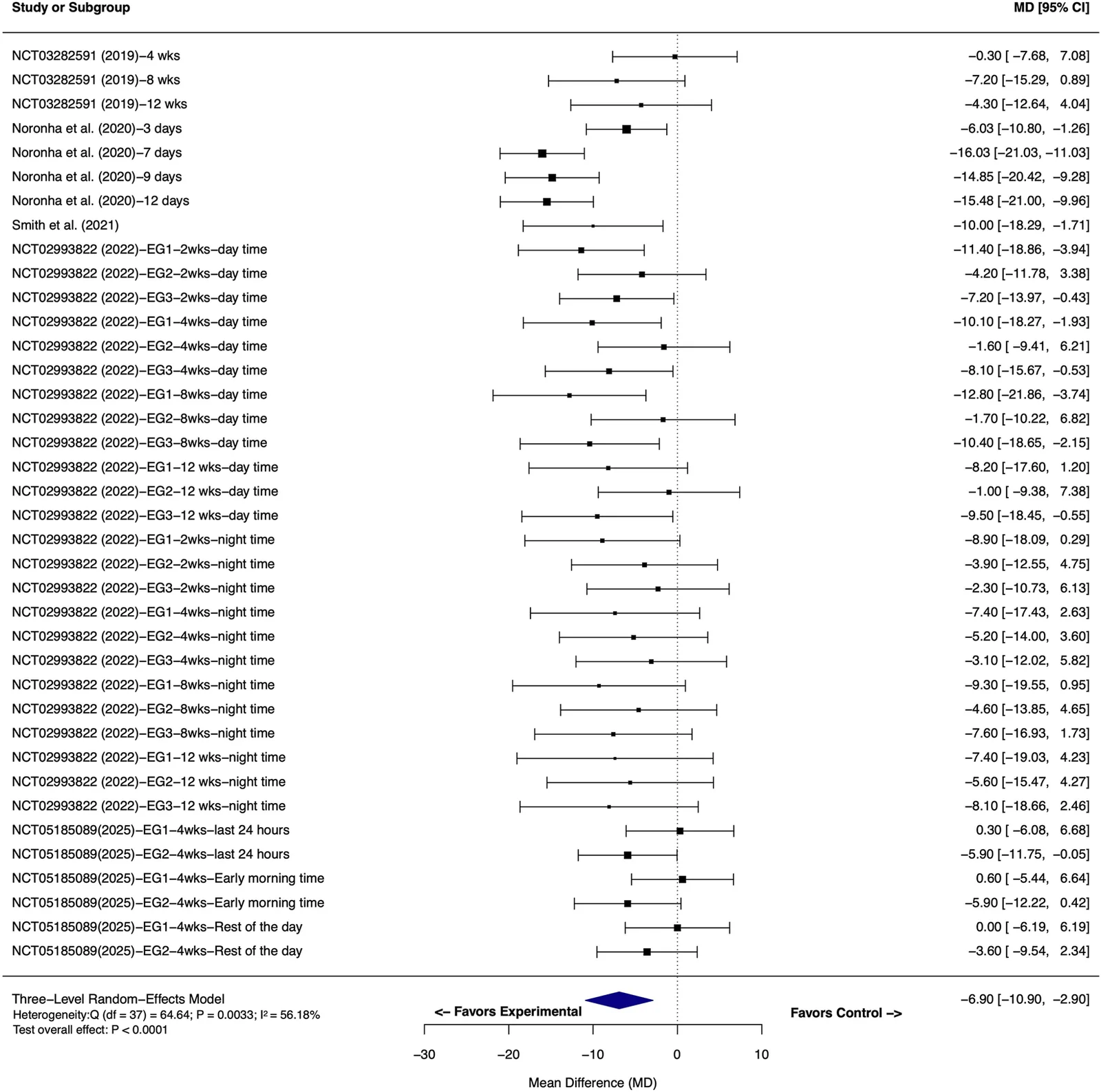
Forest plot change in efficacy for VAS.

#### 3.1.2 Cough-specific patient-reported scale

Pooled data pertaining to cough-specific patient-reported metrics revealed a statistically significant superiority in favor of the NK-1 receptor antagonist cohort compared to the control arm (SMD: 0.50, 95% CI: 0.22 to 0.78, p < 0.0001). As delineated in Figure 3, this quantitative synthesis was accompanied by substantial inter-trial heterogeneity (I^2^ = 64.5%, p = 0.0041). Clinically, these findings confirm that pharmacological intervention with NK-1 receptor antagonists substantially mitigates the overall burden of cough symptoms and drives meaningful improvements in patients’ cough-related quality of life.

**FIGURE 3:**
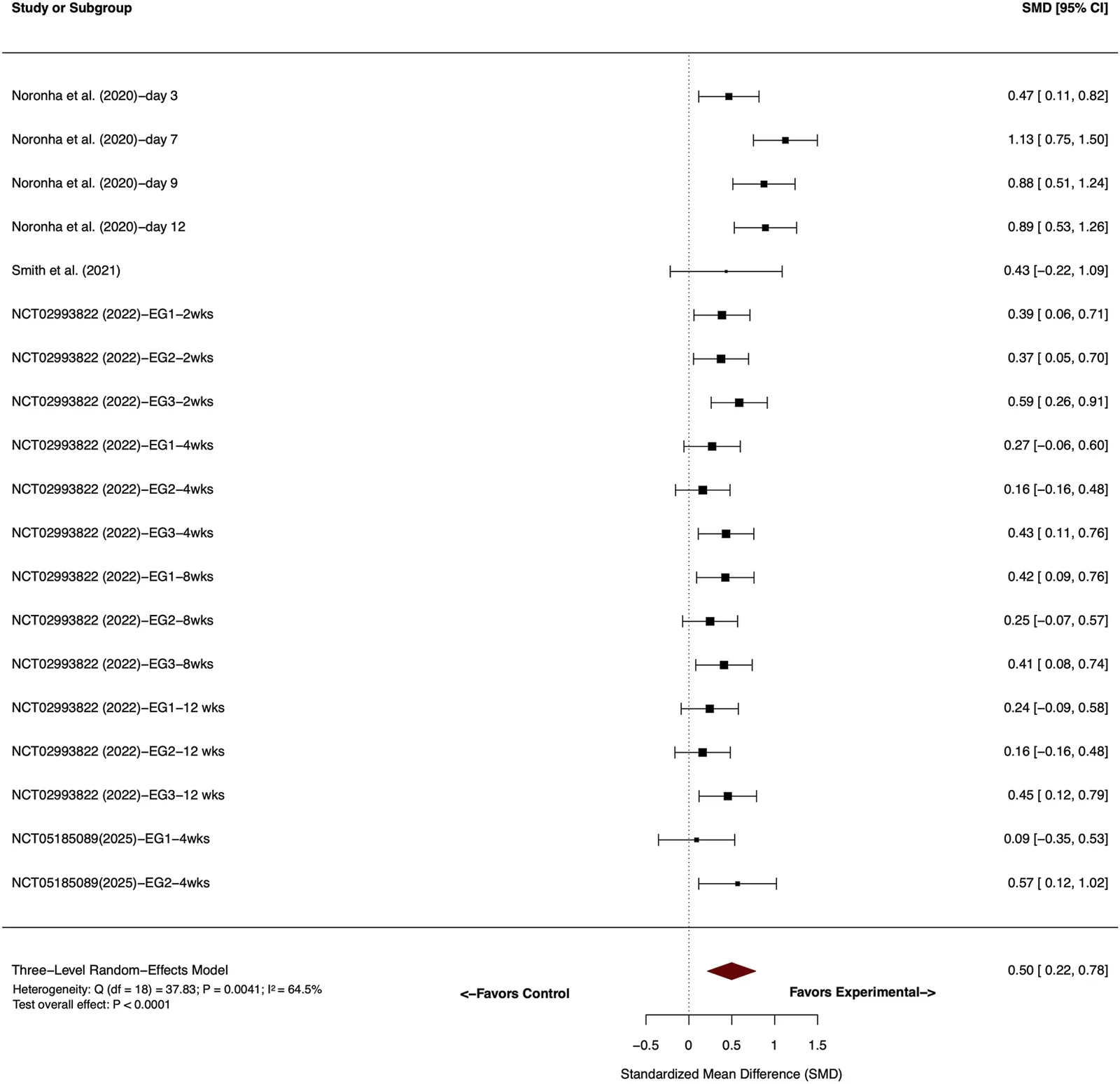
Forest plot change in cough-specific patient-reported scale.

#### 3.1.3 Cough frequency

No significant difference was detected at the overall effect estimates of awake objective cough frequency between the NK-1 receptor antagonist and placebo groups (4 studies with 15 SMD, pooled SMD: 0.01, 95% CI (−0.18, 0.21), *p* = 0.9062) and the heterogeneity is moderate (I^2^ = 42.23%, *p* = 0.2333) (Figure 4). Not statistically difference for 24 h cough frequency was found (3 studies with 4 SMD, pooled SMD: −0.08, 95% CI (−0.50, 0.34), *p* = 0.7016). The heterogeneity was high (I^2^ = 72.66%, *p* = 0.0081) (Figure 5). Not statistically difference for sleep cough frequency was detected (2 studies with 4 SMD, pooled SMD: −0.09, 95% CI (−0.43, 0.24), *p =* 0.5856). The heterogeneity was low (I^2^ = 6.9%, *p* = 0.5814) (Figure 6).

**FIGURE 4:**
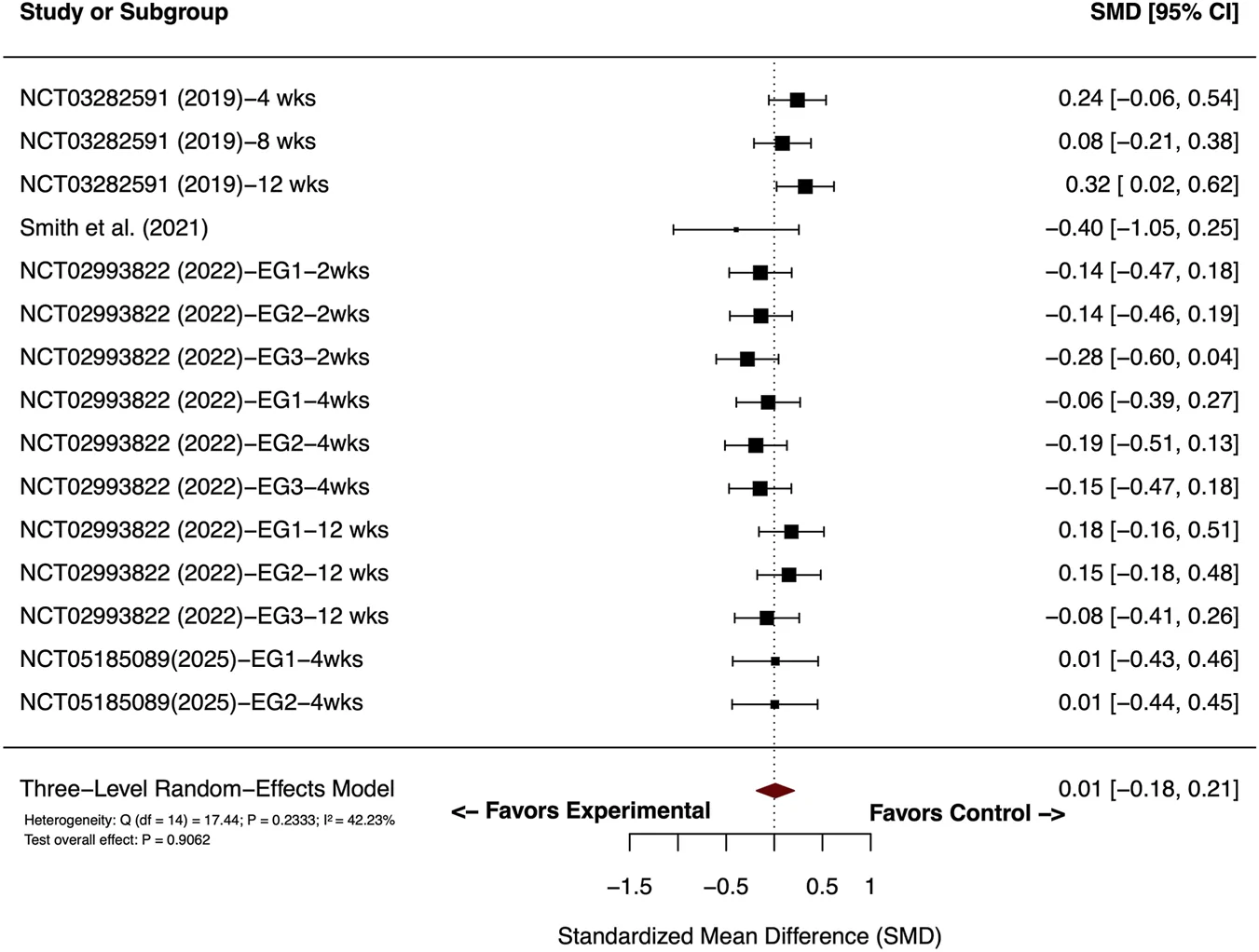
Forest plot change in efficacy for awake objective cough frequency.

**FIGURE 5:**
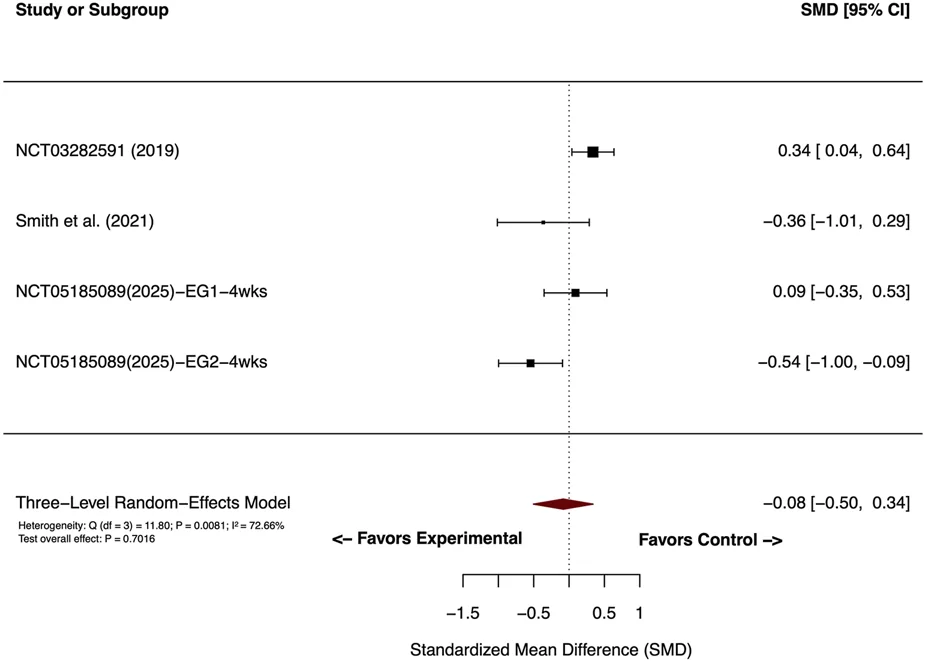
Forest plot change in efficacy for 24 h cough frequency.

**FIGURE 6:**
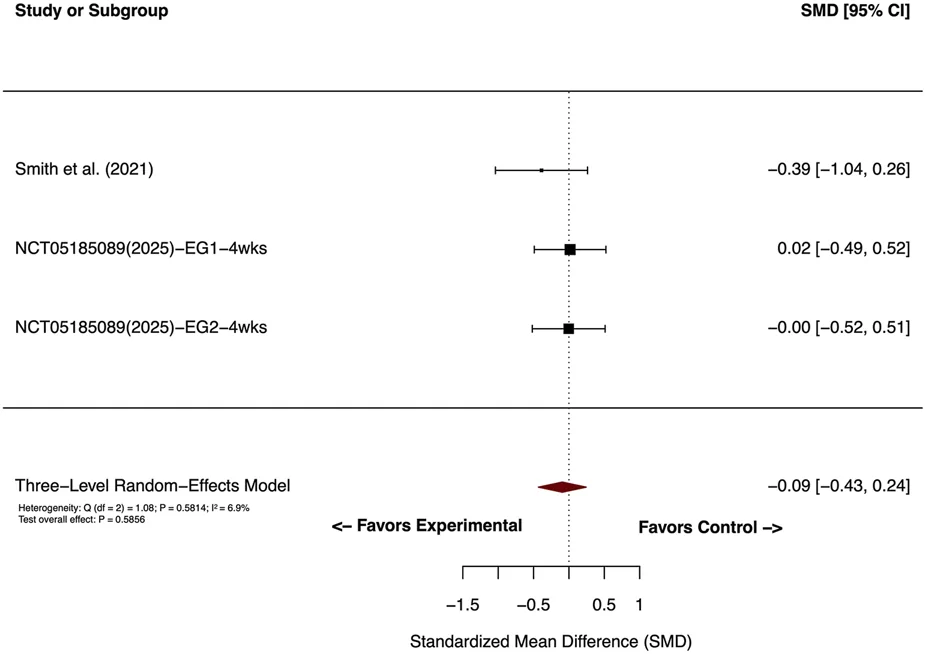
Forest plot change in efficacy for sleep cough frequency.

#### 3.1.4 Urge to cough scale

Notably, the treatment group experienced a significant reduction in the urge to cough scale (2 studies with 14 SMD, pooled SMD: −0.31, 95% CI (−0.40, −0.22), p < 0.0001) The heterogeneity was low (I^2^ = 0%, *p* = 0.5479) (Figure 7).

**FIGURE 7:**
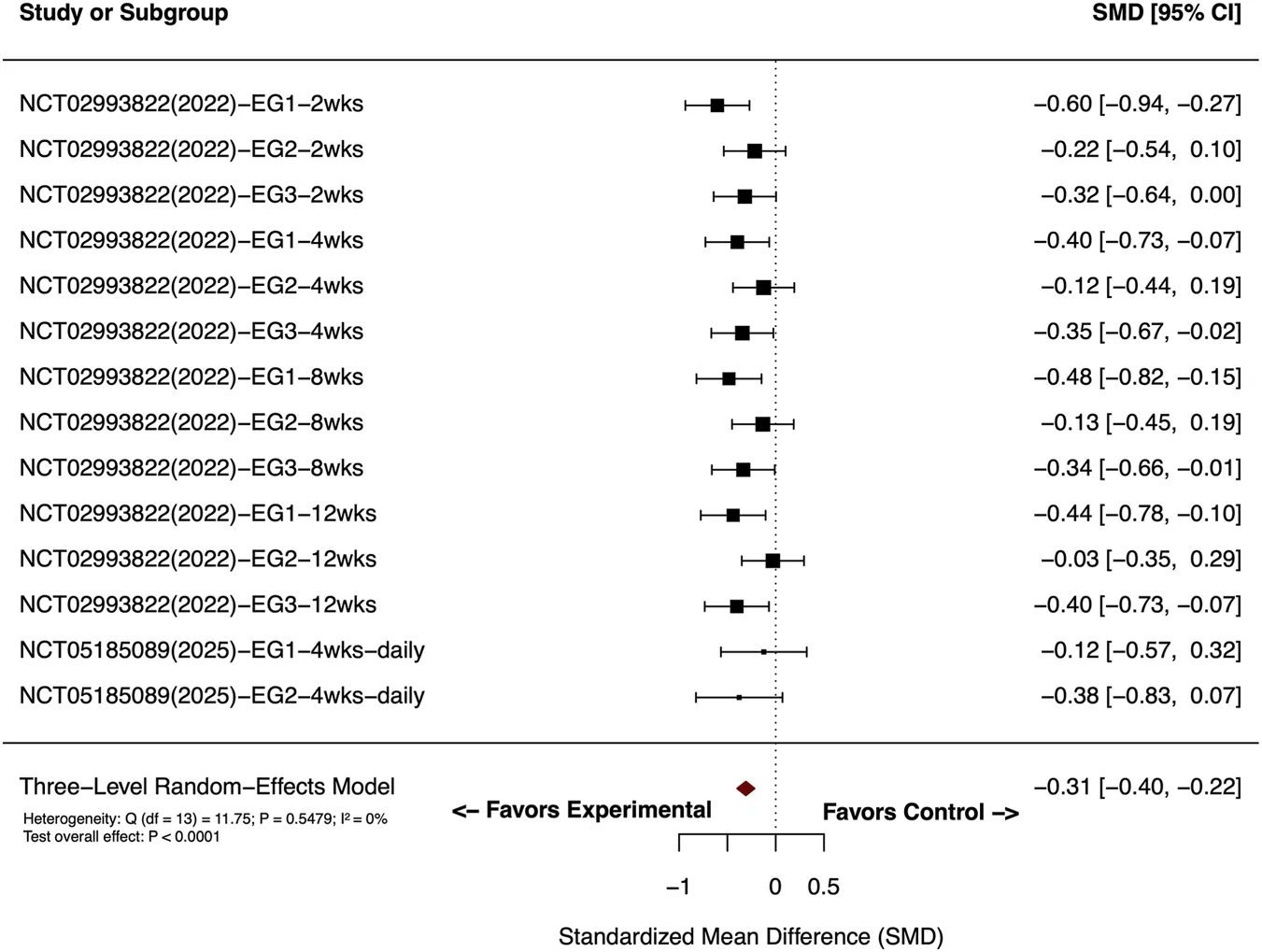
Forest plot change in efficacy for urge to cough scale.

### 3.2 Safety

Four studies assessed severe adverse events and specific adverse events, the results showed that no statistically significant difference between the treatment group and the placebo group (RR: 1.14, 95% CI (0.63, 2.07), p = 0.6578, I^2^ = 0%, p = 0.9748, Figures 8, 9). For other (not including severe) adverse events, the treatment group was significantly higher than the placebo group (RR: 1.33, 95% CI (1.15, 1.53), *p* < 0.0001; I^2^ = 42.53%, *p* = 0.0964; Figure 10). Our results for pooled effects of specific adverse events showed that somnolence, fatigue, back pain and dizziness occurred significantly in the treatment group compared with the placebo group (Figure 11).

**FIGURE 8:**
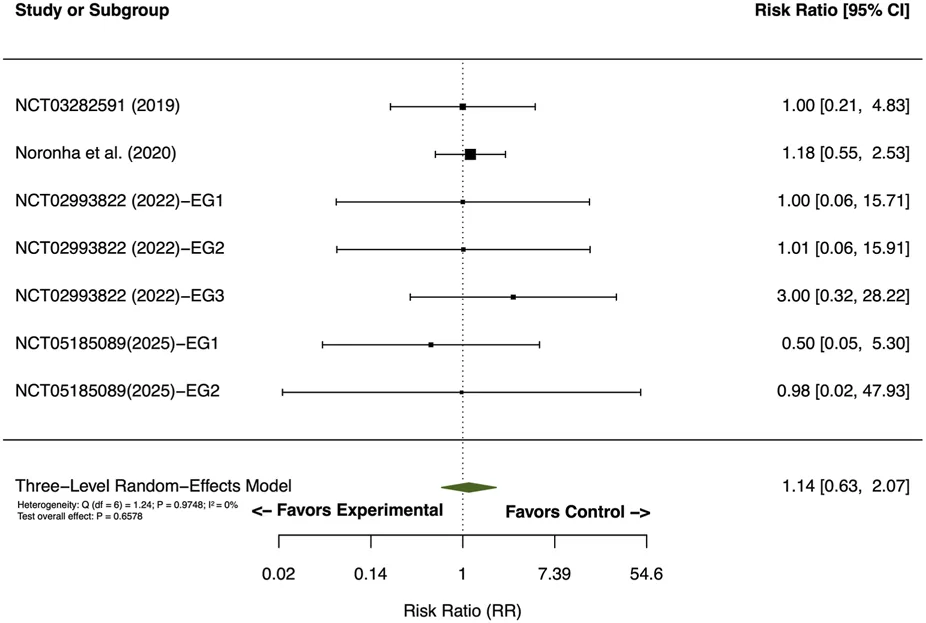
Forest plot change in safety for severe adverse events. A risk ratio greater than one indicates a higher incidence of adverse events in the treatment group.

**FIGURE 9:**
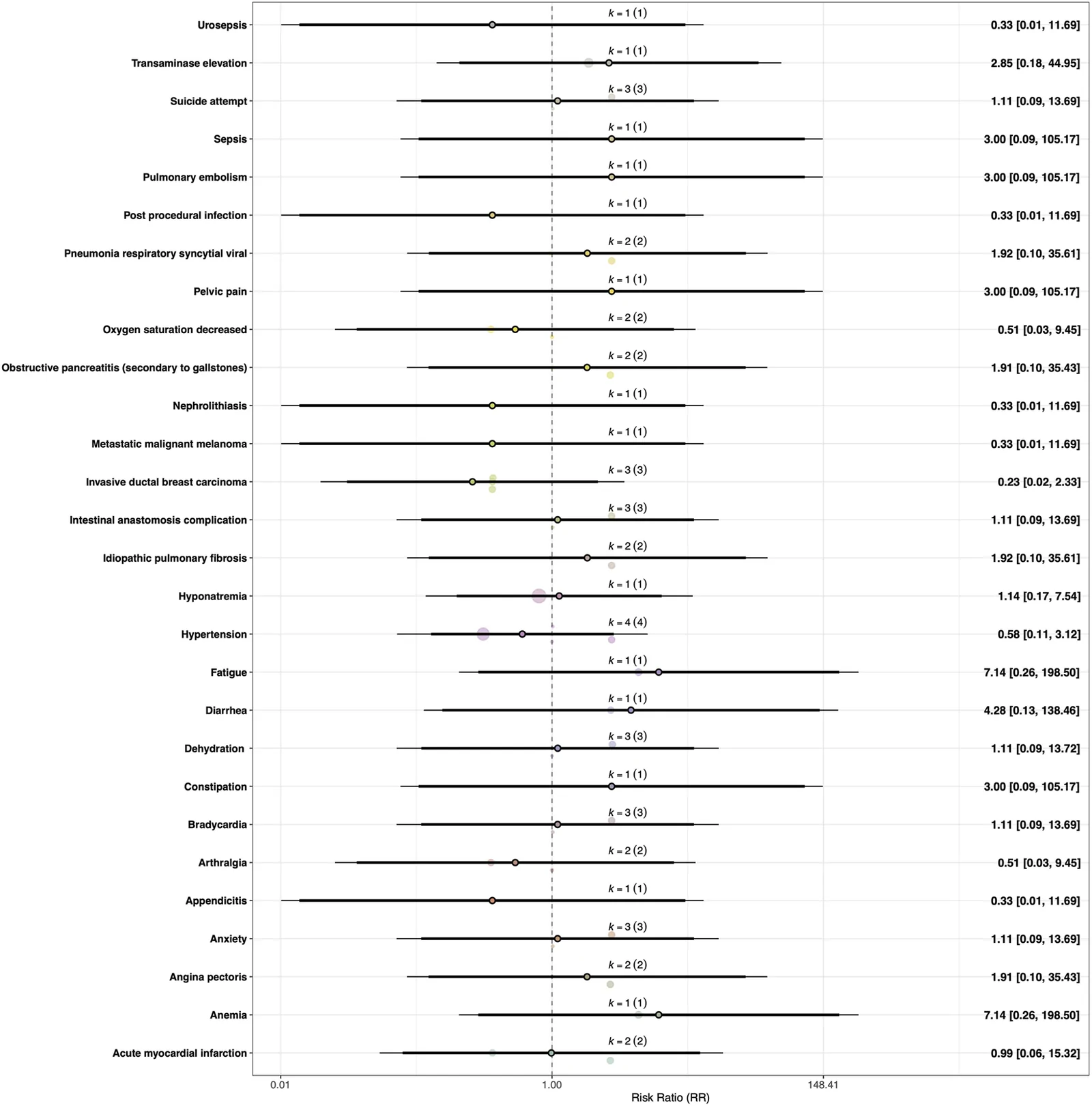
Forest plot change in safety for specific severe adverse events. A risk ratio greater than one indicates a higher incidence of adverse events in the treatment group.

**FIGURE 10:**
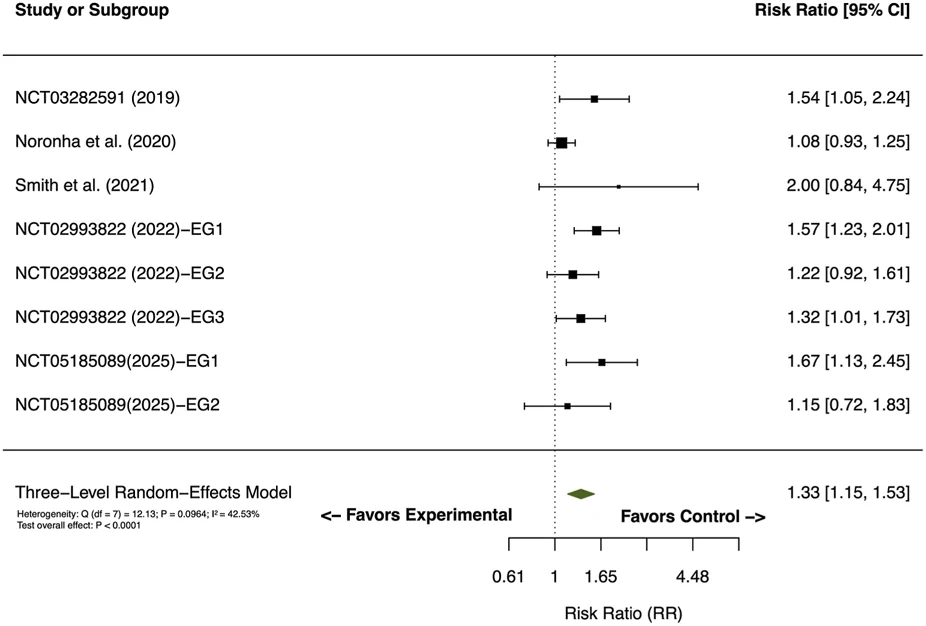
Forest plot change in safety for other (not including severe) adverse events. A risk ratio greater than one indicates a higher incidence of adverse events in the treatment group.

**FIGURE 11:**
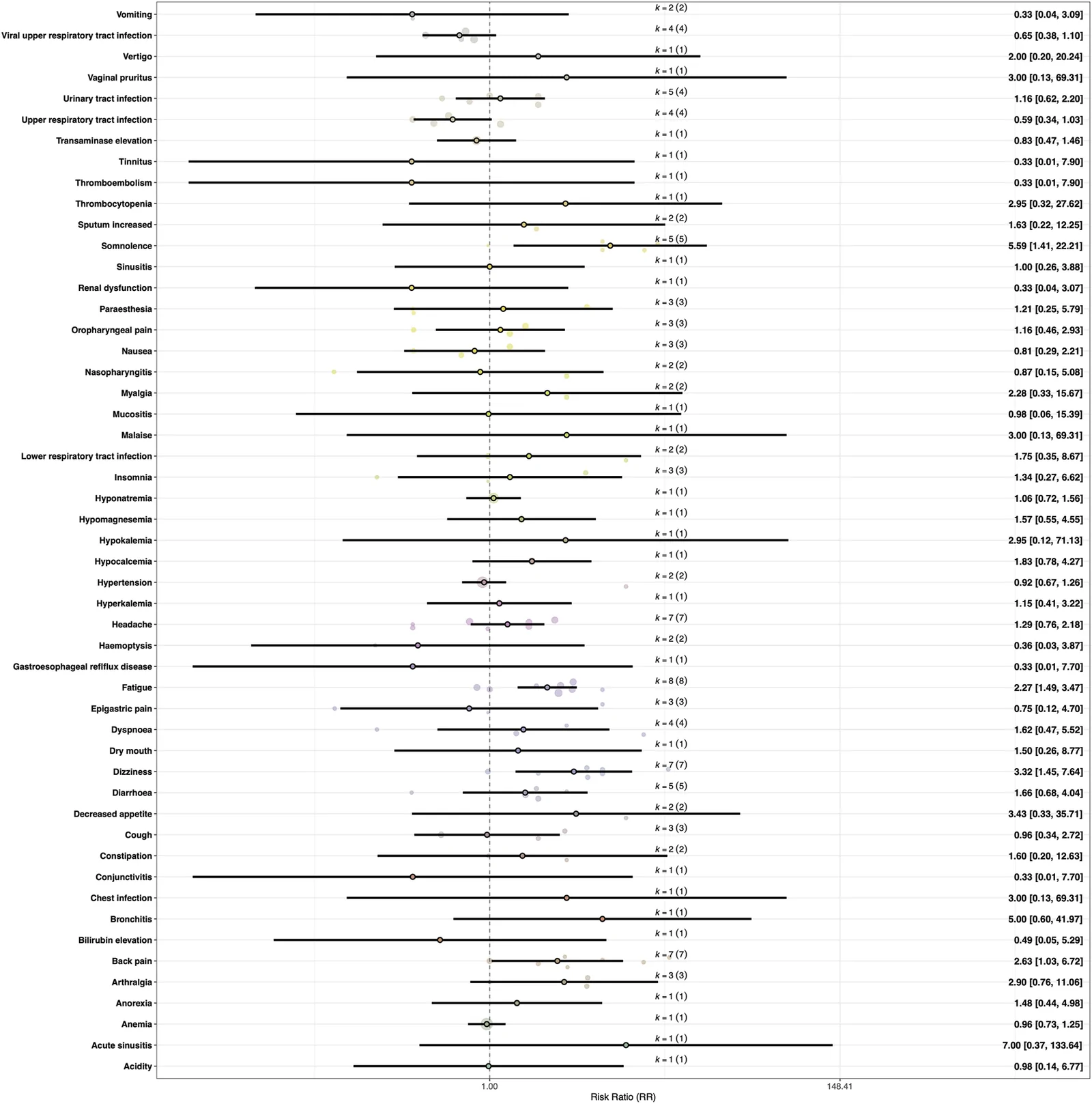
Forest plot change in safety for specific other (not including severe) adverse. A risk ratio greater than one indicates a higher incidence of adverse events in the treatment group.

### 3.3 Quality of the evidence

The certainty of evidence across all evaluated outcomes ranged from very low to low according to the GRADE framework (Supplementary Table S1). Specifically, the certainty of evidence was rated as low for awake cough frequency, severe adverse events, and non-severe adverse events, and very low for cough VAS, cough-specific patient-reported scale, 24-h cough frequency, sleep cough frequency, and urge to cough scale. The primary reasons for downgrading included clinical heterogeneity in patient populations (indirectness), potential publication bias owing to the limited number of studies (<10 RCTs), statistical heterogeneity ( $I^{2}>50\%$ , inconsistency), and small total sample sizes (<400 subjects, imprecision).

### 3.4 Sensitivity analysis

Sensitivity analysis excluding the study with a high risk of bias (Noronha et al., 2020) confirmed the robustness of our primary outcomes while substantially eliminating statistical heterogeneity across endpoints. After excluding this trial, NK-1 receptor antagonists maintained significant improvements in both cough VAS (4 trials, N = 591; MD = −4.94, 95% CI: −7.63 to −2.25; I^2^ = 19.87%, *p* = 0.6878) and cough-specific patient-reported scales (3 trials, N = 415; SMD = 0.34, 95% CI: 0.25 to 0.43; I^2^ = 0%, *p* = 0.8332). Regarding safety, no significant difference was detected for severe adverse events (3 trials, N = 571; RR = 1.09, 95% CI: 0.42 to 2.81; I^2^ = 0%, *p* = 0.9426), whereas non-severe adverse events remained significantly more frequent in the treatment group relative to placebo (4 trials, N = 591; RR = 1.42, 95% CI: 1.25 to 1.61; I^2^ = 0%, *p* = 0.6268), indicating that the high-risk study was the primary driver of between-study heterogeneity in the main synthesis (Supplementary Table S2).

## 4 Discussion

This systematic review and meta-analysis provided a comprehensive synthesis of current evidence, aiming to evaluate the efficacy and safety of NK-1 receptor antagonists in the treatment of cough. The main findings demonstrated that neurokinin-1 receptor antagonists significantly improved cough-related subjective outcomes, including cough VAS, urge to cough scale, and cough-specific patient-reported scale. However, while the statistical significance of these improvements is robust, it is important to acknowledge that the absolute magnitude of the clinical benefit remains relatively mild. Although these agents successfully modulate the sensory pathways driving the cough reflex, the modest effect sizes observed across these subjective metrics suggest they may not provide profound or complete symptom resolution for all patients. Consequently, these statistically significant results must be interpreted with careful consideration of their clinical magnitude, highlighting that NK-1 receptor antagonists may be most valuable when integrated into a multimodal therapeutic strategy or targeted toward specific patient phenotypes.

Although NK-1 receptor antagonists were associated with a higher incidence of adverse events (not including severe), particularly somnolence, fatigue, back pain and dizziness, no significant difference in severe adverse events was observed compared with placebo. These results demonstrate a generally acceptable safety profile for NK-1 receptor antagonists and support the conduct of further large-scale trials to define their efficacy and safety.

Notably, the observed benefits of NK-1 receptor antagonists were consistent across multiple subjective assessment tools, highlighting a fundamental central paradox in NK-1 receptor antagonist therapy: while treatment yields consistent clinical improvements in subjective endpoints (cough VAS, urge to cough scale, and cough-specific patient-reported scale), it fails to elicit a statistically significant reduction in objective cough frequency (awake, 24-h, or sleep counts). From a neurobiological perspective, this dissociation suggests that NK-1 receptor antagonists may primarily act by modulating central sensory processing and altering cough perception rather than directly inhibiting the brainstem motor generators of the cough reflex (Morice, 2009; Mazzone and McGarvey, 2020). NK-1 receptors are abundantly expressed in higher-order central pathways governing sensory gating, airway irritation, and the conscious urge to cough. Substance P binds to NK-1 receptors to mediate neurogenic inflammation and activate glutamatergic signalling, ultimately leading to cough hypersensitivity. (Canning et al., 2014; Smith et al., 2021). NK-1 receptor antagonists block the biological effects of substance P, which may effectively desensitize cortical awareness and attenuate the distressing sensation of cough. This mechanism potentially explains the marked improvements in patient-reported outcomes even when objective cough events persist.

In addition to neurobiological mechanisms, methodological factors may also account for this observed paradox. First, objective cough frequency measurement is subject to high intra-individual variance, meaning that small trial sample sizes may render these endpoints underpowered to detect modest reductions in physical cough counts (Smith et al., 2021). Second, robust placebo effects—well-documented in antitussive clinical trials—can substantially suppress objective cough frequency via central endogenous opioid networks, potentially obscuring true physiological drug effects between treatment and placebo arms (Eccles, 2019). Future trials utilizing standardized, continuous objective monitoring in larger, phenotyped cohorts are essential to clarify whether NK-1 blockade possesses true physiological antitussive activity or if its core therapeutic value resides in re-tuning central sensory hypersensitivity.

To our knowledge, this is the pioneering meta-analysis evaluating the pooled efficacy and safety profile of NK-1 receptor antagonists for cough treatment. Despite this strength, several key limitations must be explicitly acknowledged. First and foremost, a major conceptual constraint of our study is the pooling of patient populations across distinct clinical entities—namely, refractory chronic cough, lung cancer-associated cough, and idiopathic pulmonary fibrosis (IPF)-associated cough. Although these conditions may overlap in downstream sensory hypersensitivity, their primary etiologies, underlying tissue pathologies, and neuro-inflammatory mechanisms are fundamentally different. Consequently, NK-1 receptor antagonists cannot be assumed to possess a uniform, disease-agnostic class effect across distinct cough pathologies, and our pooled effect estimates must be interpreted with extreme caution. Given that only five trials met our eligibility criteria, formal subgroup analyses or meta-regression by disease etiology were statistically unfeasible, in accordance with Cochrane recommendations stipulating a minimum of 10 studies to prevent spurious inferences (Deeks et al., 2019). Therefore, our quantitative synthesis should be regarded strictly as an exploratory aggregation of the current sparse literature rather than evidence of universal therapeutic efficacy.

Second, the small evidence base (five trials, 719 participants) incorporates substantial methodological variability, including distinct pharmacological agents (aprepitant, orvepitant, and serlopitant), varied dosages, treatment durations ranging from days to weeks, and combined parallel and crossover study designs. Lastly, outcome assessment lacked standardization; trials utilized distinct subjective clinical scales, while objective cough frequency monitoring was inconsistently quantified. Consequently, standardized mean differences (SMD) were utilized to synthesize heterogeneous endpoints, but future larger-scale, phenotype-stratified randomized controlled trials are strictly required to determine whether true therapeutic utility exists within specific patient subpopulations.

## 5 Conclusion

Our systematic review and meta-analysis suggest that NK-1 receptor antagonists provide modest improvements in patient-reported subjective cough outcomes including cough VAS, urge to cough scale and cough-specific patient-reported outcomes. However, these agents demonstrate no statistically significant reduction in objectively measured cough frequency, and confidence in the pooled estimates is substantially limited by the small evidence base and the combining of etiologically distinct clinical entities (such as refractory chronic cough, IPF, and lung cancer). Large-scale, high-quality randomized controlled trials are needed to evaluate their therapeutic efficacy, optimal target populations, and safety.

## Data Availability

The original contributions presented in the study are included in the article/Supplementary Material, further inquiries can be directed to the corresponding authors.
