# Supplementary material for: Efficacy and safety of neurokinin-1 receptor antagonist on cough suppression: a systematic review and meta-analysis
Source: Front Pharmacol. 2026 Oct 1;17:1915023. doi: 10.3389/fphar.2026.1915023 (PMC13670168; doi:10.3389/fphar.2026.1915023)
Supplement: Supplementary file 1 [file Table1.docx]

**eTable 1: The results of quality of the evidence**

| **Certainty assessment** | | | | | | | **№ of patients** | | **Effect** | | **Certainty** | **Importance** |
| --- | --- | --- | --- | --- | --- | --- | --- | --- | --- | --- | --- | --- |
| **№ of studies** | **Study design** | **Risk of bias** | **Inconsistency** | **Indirectness** | **Imprecision** | **Other considerations** | **[NK-1]** | **[Placebo]** | **Relative (95% CI)** | **Absolute (95% CI)** |  |  |
| **Cough visual analog scale (VAS)** | | | | | | | | | | | | |
| 5 | randomised trials | not serious | serious^a^ | serious^b^ | not serious | publication bias strongly suspected^c^ | 438 | 281 | - | MD **6.9 SD lower** (10.9 lower to 2.9 lower) | ⨁◯◯◯ Very low^a,b,c^ | IMPORTANT |
| **Cough-specific patient-reported scale** | | | | | | | | | | | | |
| 4 | randomised trials | not serious | serious^a^ | serious^b^ | not serious | publication bias strongly suspected^c^ | 350 | 193 | - | SMD **0.5 SD higher** (0.22 higher to 0.78 higher) | ⨁◯◯◯ Very low^a,b,c^ | CRITICAL |
| **Cough frequency** | | | | | | | | | | | | |
| 4 | randomised trials | not serious | not serious | serious^b^ | not serious | publication bias strongly suspected^c^ | 374 | 217 | - | SMD **0.01 SD higher** (0.18 lower to 0.21 higher) | ⨁⨁◯◯ Low^b,c^ | CRITICAL |
| **24 h cough frequency** | | | | | | | | | | | | |
| 3 | randomised trials | not serious | serious^a^ | serious^b^ | serious^d^ | publication bias strongly suspected^b,c^ | 138 | 138 | - | SMD **0.08 SD lower** (0.5 lower to 0.34 higher) | ⨁◯◯◯ Very low^a,b,c,d^ | IMPORTANT |
| **Sleep cough frequency** | | | | | | | | | | | | |
| 2 | randomised trials | not serious | not serious | serious^b^ | serious^d^ | publication bias strongly suspected^c^ | 50 | 50 | - | SMD **0.09 SD lower** (0.43 lower to 0.24 higher) | ⨁◯◯◯ Very low^b,c,d^ | IMPORTANT |
| **Urge to cough** | | | | | | | | | | | | |
| 2 | randomised trials | not serious | not serious | serious^b^ | serious^d^ | publication bias strongly suspected^c^ | 276 | 119 | - | SMD **0.31 SD lower** (0.43 lower to 0.24 higher) | ⨁◯◯◯ Very low^b,c,d^ | IMPORTANT |
| **Severe adverse events** | | | | | | | | | | | | |
| 4 | randomised trials | not serious | not serious | serious^b^ | not serious | publication bias strongly suspected^c^ | 21/470 (4.5%) | 6/311 (1.9%) | **RR 1.14** (0.63 to 2.07) | **3 more per 1,000** (from 7 fewer to 21 more) | ⨁⨁◯◯ Low^b,c^ | CRITICAL |
| **Adverse events (not including severe)** | | | | | | | | | | | | |
| 5 | randomised trials | not serious | not serious | serious^b^ | not serious | publication bias strongly suspected^c^ | 451/781 (57.7%) | 262/585 (44.8%) | **RR 1.33** (1.15 to 1.53) | **148 more per 1,000** (from 67 more to 237 more) | ⨁⨁◯◯ Low^b,c^ | CRITICAL |

**CI:** confidence interval; **MD:** mean difference; **RR:** risk ratio; **SMD:** standardised mean difference

#### Explanations

a. Substantial heterogeneity exists (I² > 50%).

b. The population included in the RCTs is not homogeneous (RCC, cough due to IPF or lung cancer)

c. Strict assessment was not possible due to fewer than 10 studies; the rating was downgraded directly.

d. Fewer than 400 subjects were included in the meta-analysis

**eTable 2:** **Summary of sensitivity analysis results**

| **Outcomes** | **Trials (No.Sample)** | **Effect size (95% CI)** | **I2 (p value)** | **Comparison with the original result** |
| --- | --- | --- | --- | --- |
| Cough visual analog scale (VAS) | 4 (591) | -4.94 (-7.63, -2.25) | 19.87% (0.6878) | Heterogeneity decreased markedly, and the effect size remained statistically significant. |
| Cough-specific patient-reported scale | 3 (415) | 0.34 (0.25, 0.43) | 0% (0.8332) | Heterogeneity decreased markedly, and the effect size remained statistically significant. |
| Severe adverse events | 3 (571)) | 1.09 (0.42, 2.81) | 0% (0.9426) | No significant changes in heterogeneity or effect size. |
| Other (not including severe) adverse events | 4 (591) | 1.42 (1.25, 1.61) | 0% (0.6268) | Heterogeneity decreased markedly, and the effect size remained statistically significant. |
